# Supplementary material for: Association between Vitamin D Deficiency and Clinical Parameters in Men and Women Aged 50 Years or Older: A Cross-Sectional Cohort Study
Source: Nutrients. 2023 Jul 5;15(13):3043. doi: 10.3390/nu15133043 (PMC10346261; doi:10.3390/nu15133043)
Supplement: Supplementary file 1 [file nutrients-15-03043-s001.zip › nutrients-2475935-supplementary.pdf]

Supplementary TableS1. Multiple linear regression analyses for 25(OH)D as outcome in women

Variable	Model 1		Model2		Model 3	
	β (SE)	*p*-Value	β (SE)	*p*-Value	β (SE)	*p*-Value
HbA1c	-3.267 (1.246)	0.010	-2.777 (1.240)	0.027	-2.672 (1.166)	0.024
BMI	-0.877 (0.270)	0.002	-0.772 (0.270)	0.005	-0.611 (0.260)	0.020
TNF-α	-1.394 (0.601)	0.022	-1.382 (0.582)	0.019	-1.188 (0.552)	0.033
IL-6	-0.322 (0.672)	0.633	-0.175 (0.662)	0.792	-0.434 (0.623)	0.487
IL-1β	-0.191 (0.803)	0.813	0.083 (0.793)	0.917	0.374 (0.769)	0.628
HGS	0.252 (0.271)	0.354	0.226 (0.268)	0.402	0.138 (0.253)	0.585
CST	0.225 (0.367)	0.542	0.067 (0.363)	0.853	-0.129 (0.352)	0.714

Data are expressed as the Beta-coefficient (β) and standard error (SE). BMI: body mass index; HGS: handgrip strength; CST: chair stand test. Model 1: adjusted for age. Model 2: adjusted for age, smoking, alcohol drinking, and exercise. Model 3: adjusted for age, smoking, alcohol drinking, exercise, eGFR, diabetes, hypertension, and vitamin D replacement.

Supplementary TableS2. Correlations between 25(OH)D levels and clinical parameters in men and women

	**25(OH)D**			
	Men		Women	
Variables	*r*	*p*-Value	*r*	*p*-Value
Age (years)	-0.029	0.623	-0.021	0.816
BMI (kg/m^2)	-0.014	0.815	-0.282	0.001
HbA1c (%)	-0.087	0.141	-0.231	0.010
TNF-α (pg/mL)	-0.035	0.551	-0.206	0.021
IL-6 (pg/mL)	0.083	0.158	-0.044	0.625
IL-1β (pg/mL)	0.010	0.864	-0.020	0.824
HGS (kg)	0.069	0.244	0.086	0.342
CST (sec)	0.106	0.076	0.058	0.542

Values are Pearson’s correlation coefficients. BMI: body mass index; HGS: handgrip strength; CST: chair stand test.
